# Supplementary material for: NOX4 functions as a mitochondrial energetic sensor coupling cancer metabolic reprogramming to drug resistance
Source: Nat Commun. 2017 Oct 19;8:997. doi: 10.1038/s41467-017-01106-1 (PMC5648812; doi:10.1038/s41467-017-01106-1)
Supplement: Supplementary file 1 — Supplementary Information [file 41467_2017_1106_MOESM1_ESM.pdf]

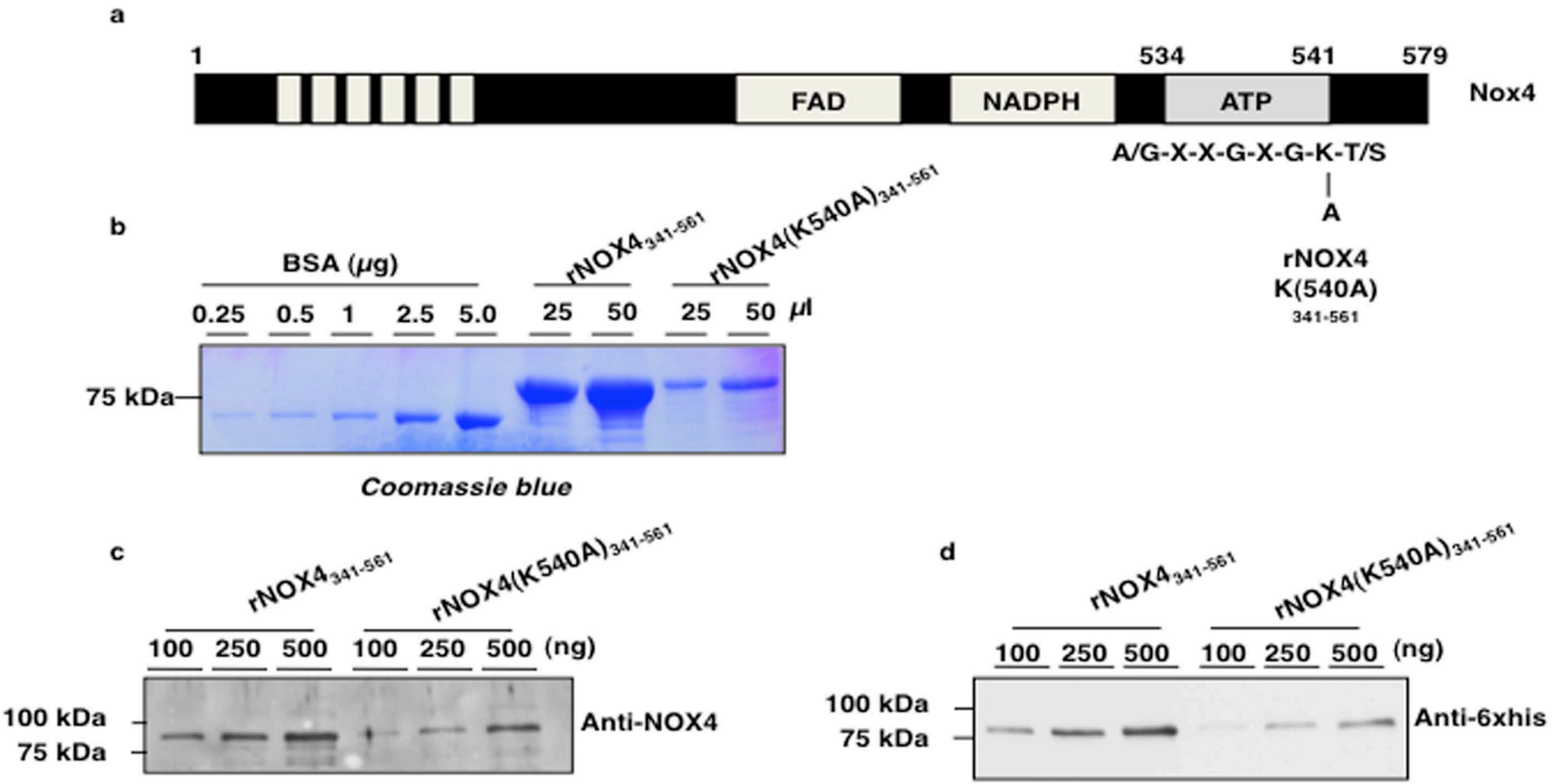

Supplementary Figure 1. Purification and characterization of NOX4 recombinant protein. (**a**) Cartoon of full-length NOX4 highlighting Walker A ATP binding motif within amino acids 534-541. The critical lysine residue necessary for ATP binding (K540) was mutated using site-directed mutagenesis to alanine to generate, NOX4 (K540A). (**b**) hNOX4 cDNA (a.a. 341-561) was cloned into a NUSA/6xHIS vector system to generate recombinant protein. WT NOX4 341-561 and MUT NOX4 341-561, K540A proteins were purified using NI-NTA beads and increasing amounts of recombinant (r) proteins were resolved on SDS-PAGE and stained with coomassie blue to demonstrate purity. BSA was included for quantitation. (**c,d**) Increasing amounts of $rNOX4^{341-561}$ and NOX4 $(K540A)^{341-561}$ purified proteins were resolved on two-independent SDS-PAGE gels and Western blot analysis was performed using (**c**) NOX4 antibodies (Lab) and (**d**) 6xHIS (right panel).

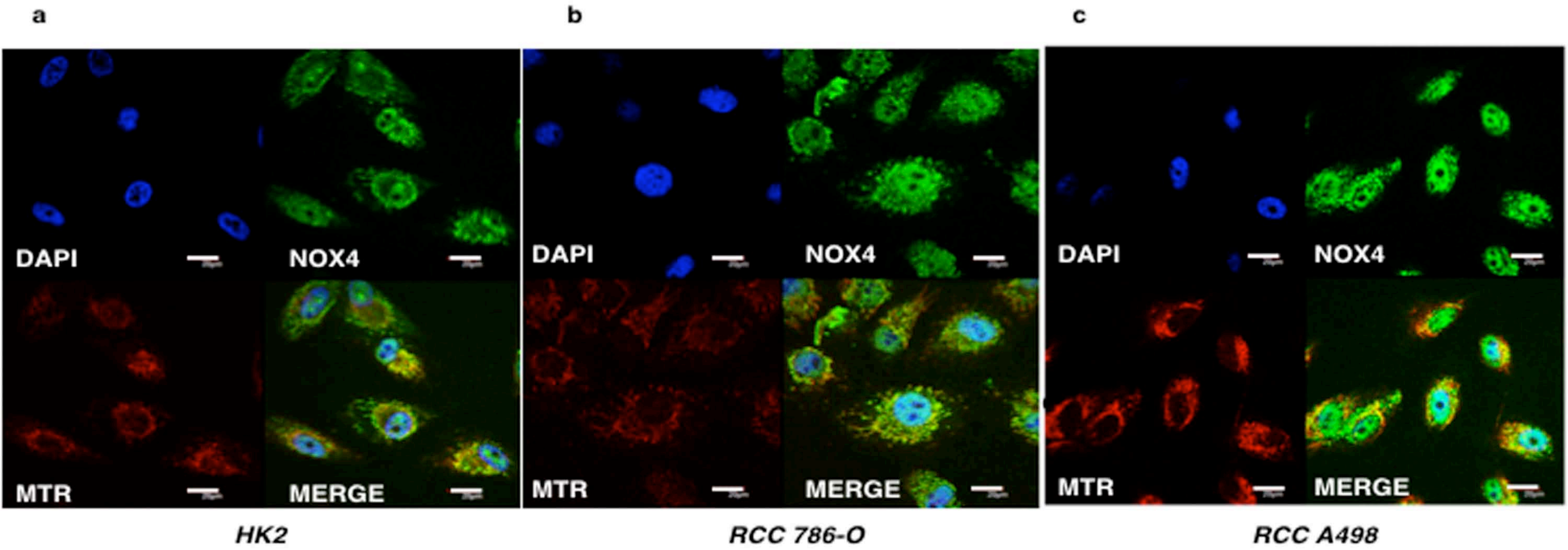

Supplementary Figure 2. NOX4 localizes to the mitochondria. Subcellular localization of NOX4 was assessed in normal renal epithelial cells, (**a**) HK2 and von Hippel Lindau (VHL)-deficient renal carcinoma cells (RCC) (**b**) 786-O and (**c**) A498 by confocal microscopy as described (8). Indicated renal cells were labeled with mitotracker red (MTR), fixed, and stained with NOX4 (Novus49) antibody followed by FITC-linked donkey anti-rabbit secondary antibody. Nuclei were counter stained with DAPI. Merge in yellow shows colocalization. Scale bars: 20μM.

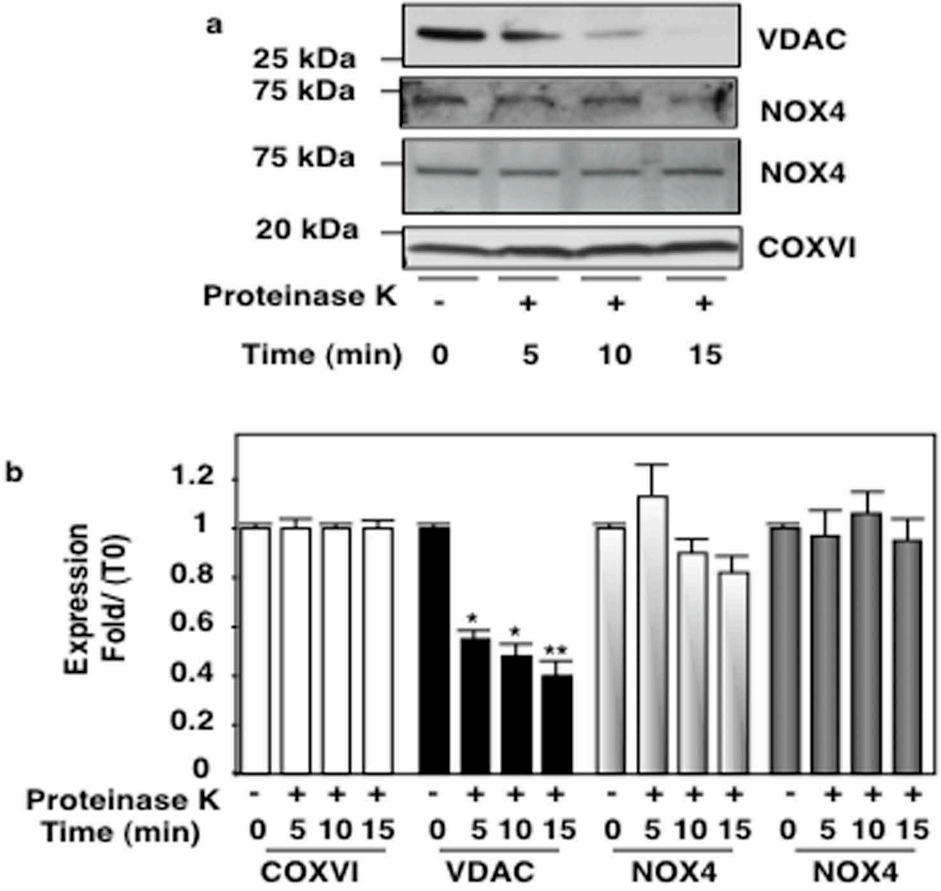

Supplementary Figure 3. NOX4 localizes to the inner mitochondrial membrane. (**a**) Percoll gradient purified mitochondria fraction was prepared from rat kidney cortex and equal amounts of protein were subjected (+) or not (-) to proteinase K digestion over time (T0, T5, T10, and T15 min). VDAC, COX VI, and NOX4 (Novus 49 upper panel, our NOX4 antibody lower panel) were analyzed by Western blot analysis. (**b**) Quantitation of COXVI, VDAC, and NOX4 expression. The results are expressed as the means using one-way ANOVA with Tukey's post hoc test where ± S.E.M *$p<0.05$, **$p<0.01$, compared to non-treated control.

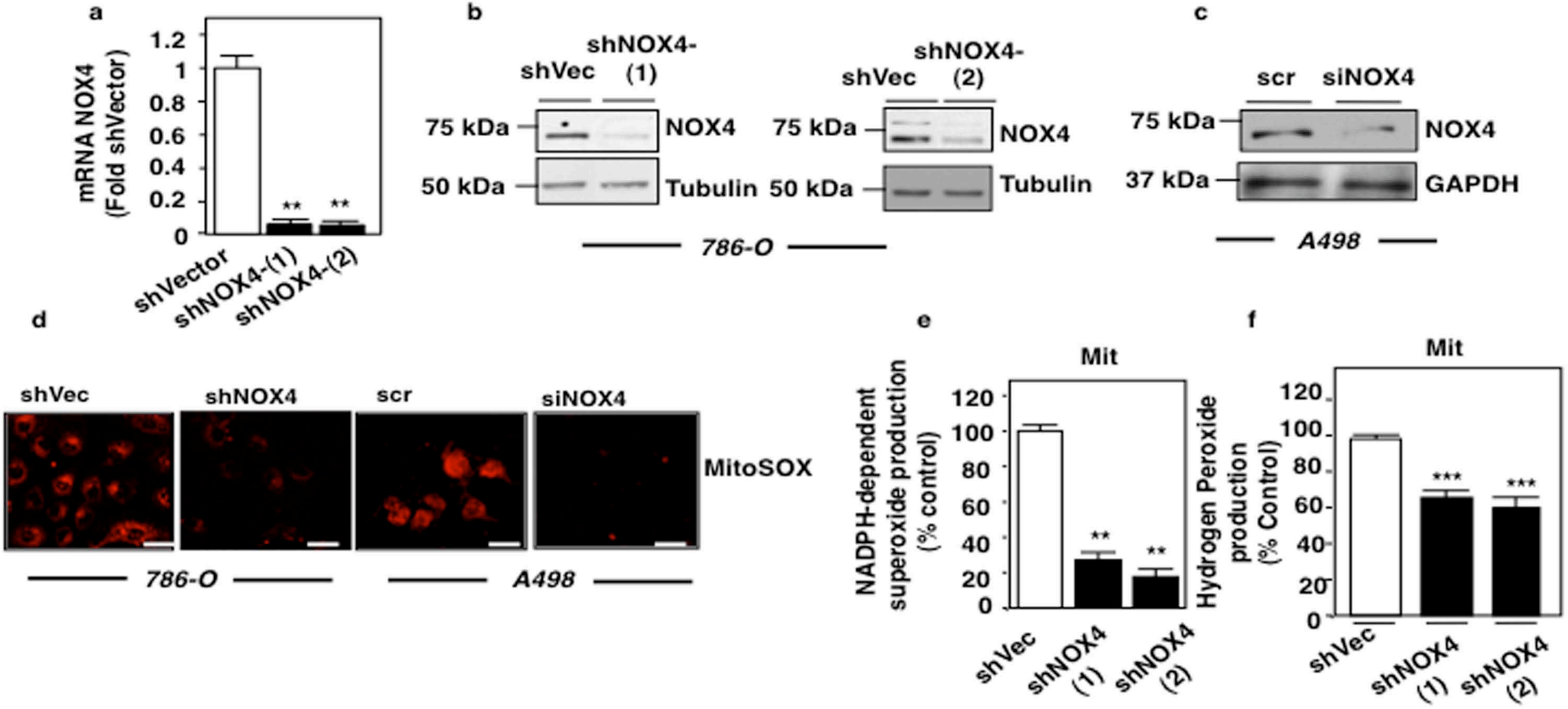

Supplementary Figure 4. Generation and characterization of stable and transient NOX4 knockdown. (**a**) NOX4 mRNA was examined using qRT-PCR in stable lentiviral NOX4 knockdown cells compared to vector control. Values are the means ± SE from three independent experiments using one-way ANOVA with Tukey's post hoc test where **, $p < 0.01$ versus shVector cells (**b**) NOX4 protein expression was examined in the stable shVector and shNOX4 single cell clones or (**c**) A498 cells using Western blot analysis siNOX4 knockdown. (**d**) (Upper panel) RCC 786-O or A498 cells were transfected with shRNA or siRNA against NOX4 respectively in parallel, shVec or scrambled (scr) controls were transfected as controls. 48 hrs post-transfection, transfected cells were exposed to MitoSOX, a fluorogenic dye specifically targeted to mitochondria in live cells. Representative images obtained by confocal fluorescence microscopy are indicated. Bars 20μM (Lower panel) Bright field of upper panel. (**e**) NADPH-dependent superoxide generation was measured by enhanced chemiluminescence in mitochondrial fractions of shVector and shNox4 stable RCC-786-O cells. (**f**) Hydrogen peroxide was measured in mitochondria fraction of shVector and shNox4 stable RCC-786-O cells using Amplex Red reagent.

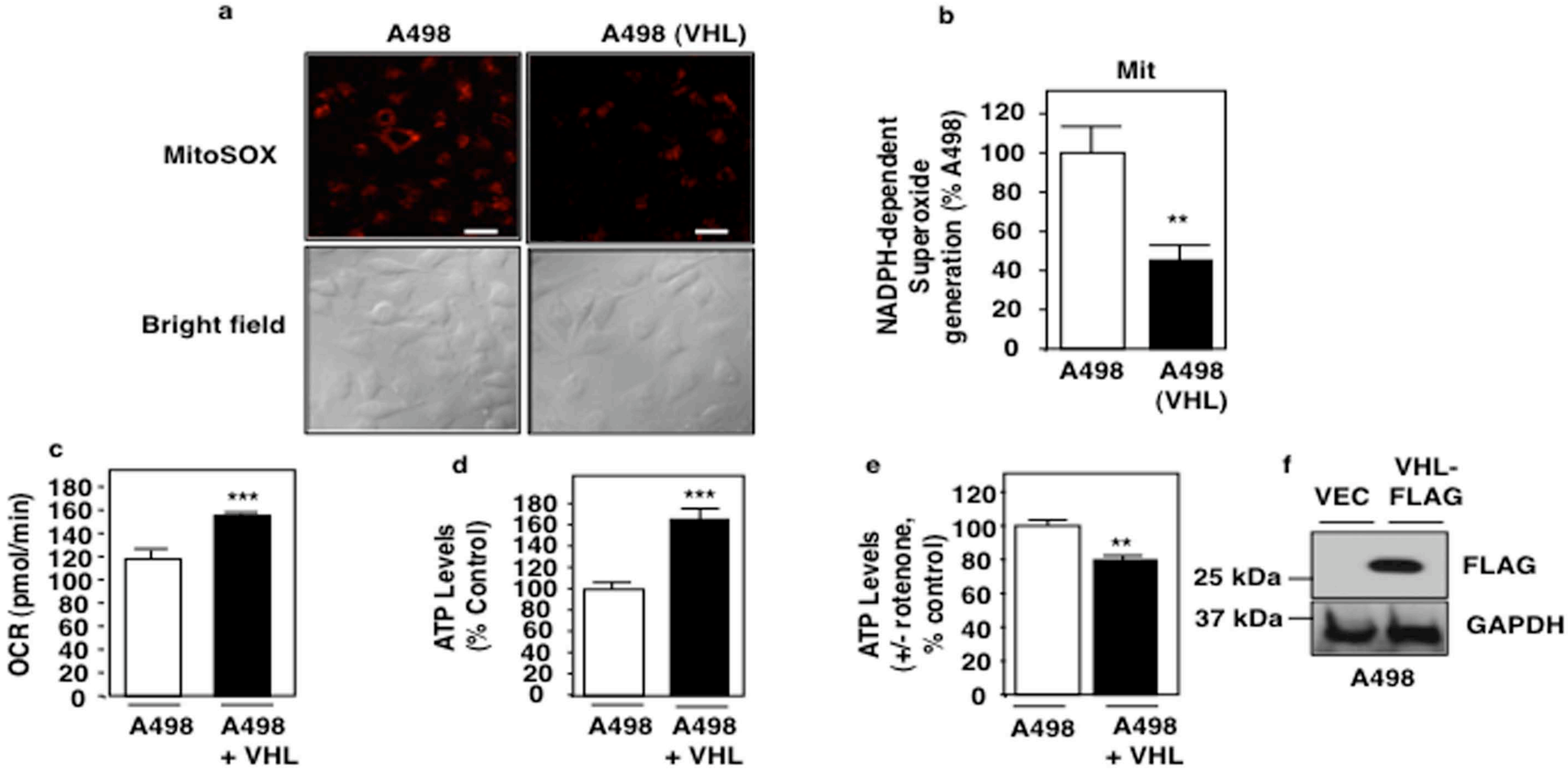

Supplementary Figure 5. Loss of VHL in RCC A498 cells correlates with reduced ATP levels and enhanced NOX activity, which is reversed upon VHL re-introduction. RCC A498 cells were transfected with or without VHL using lipofectamine. The cells were plated and assessed 24 hours post-transfection for the indicated outputs in 2-3 independent experiments: (**a**) (Upper panel) Superoxide detection within the mitochondria was assessed in 2-independent experiments using MitoSOX and confocal microscopy Bars 20µM. (Lower panel) Bright field of upper panel. (**b**) NADPH oxidase activity was evaluated and expressed as the means of 3-independent experiments using one-way ANOVA with Tukey's post hoc test where ± S.E.M $^{**}p<0.01$, compared to transfected A498 cells without VHL. (**c**) Oxygen consumption (pmol/min) was measured in live cells using Seahorse technology and expressed as the means using one-way ANOVA with Tukey's post hoc test where ± S.E.M $^{***}p<0.001$, compared to transfected A498 cells without VHL from 2-independent experiments of 4 wells each. (**d**) Total ATP levels were measured without (**d**) or with (**e**) 1uM rotenone. The results are from 3 independent experiments and expressed as the means using one-way ANOVA with Tukey's post hoc test where ± S.E.M $^{**}p<0.01$, $^{***}p<0.001$ compared to transfected A498 cells without VHL. (**f**) Successful reintroduction of VHL was verified by Western blot analysis using anti-Flag antibodies.

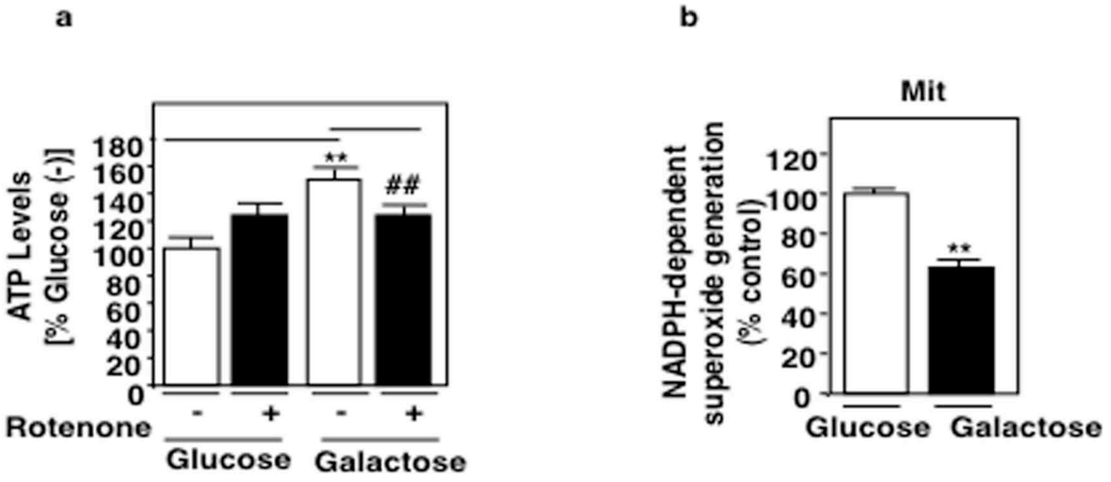

Supplementary Figure 6. Effects of Galactose on ATP levels and NOX activity in RCC cells. (**a**) RCC 786-O cells cultured in glucose or galactose for 24 hrs time were exposed 30 min (+) or not (-) to rotenone (1uM) and ATP levels were measured as outlined in methods. The results are from 3-independent experiments and expressed as the means using one-way ANOVA with Tukey's post hoc test where ± S.E.M **p<0.01, compared to glucose cultured cells exposed to buffer (-) alone or ± S.E.M ##p<0.01 compared to galactose cultured cells exposed to buffer control (-). (**b**) Mitochondrial fractions were prepared from RCC 786-O cells cultured in glucose and galactose and NADPH-dependent superoxide generation was examined by chemiluminescence. The results are from 3-independent experiments and expressed as the means using one-way ANOVA with Tukey's post hoc test where ± S.E.M **p<0.01, compared to glucose cultured cells.

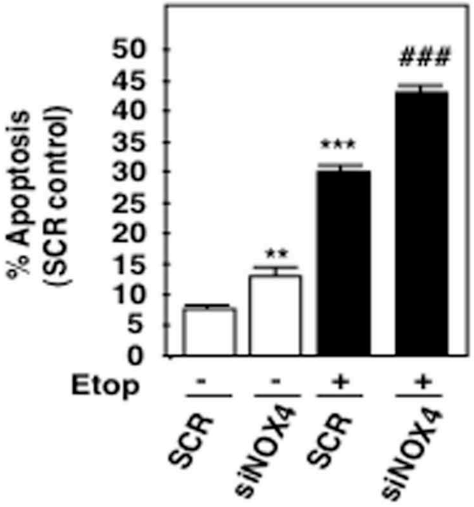

Supplementary Figure 7. Effects of NOX4 silencing on drug-induced cell death in VHL-deficient A498 cells. RCC A498 cells were transfected (Amaxa) with scrambled control (SCR) or small inhibitory RNA against NOX4 (siNOX4). After 24 hours, transfected cells were incubated (+) or not (-) with 100 μM Etoposide for 14 hrs. Apoptosis was analyzed by Annexin V staining and flow cytometry.

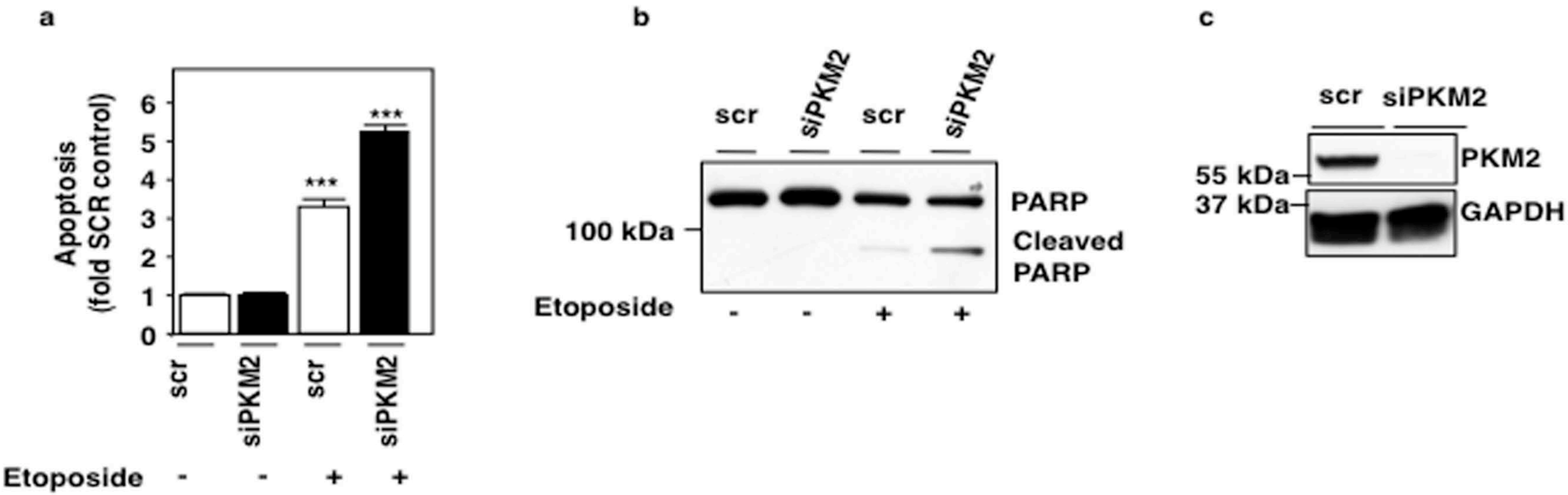

Supplementary Figure 8. Effects of PKM2 silencing on drug-induced cell death in A498 RCC cells. Scrambled (scr) or siRNA against PKM2 (siPKM2) was transfected in RCC A498 cells. After 48 hours, transfected (Amaxa) cells were incubated (+) with or without (-), etoposide (100 μM) for 14 hours. (**a**) Apoptosis was analyzed by Annexin V staining and flow cytometry. The results are presented from at least 3-independent experiments and expressed as the means using one-way ANOVA with Tukey's post hoc test where ± S.E.M ***p<0.001 compared to respective controls not exposed to etoposide. (**b**) In parallel, cell lysates were prepared and PARP cleavage was examined by Western blot analysis. (**c**) PKM2 Western blot analysis shows successful PKM2 downregulation in A498 cells. GAPDH was used as a loading control.

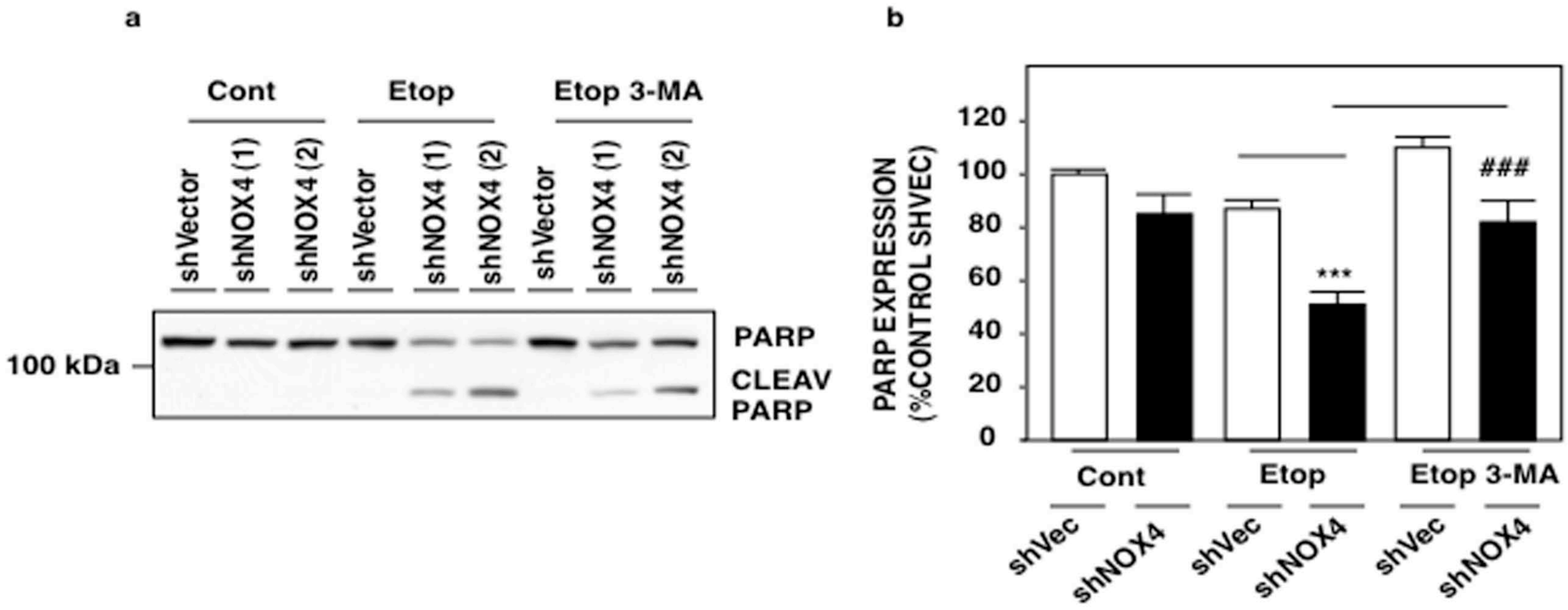

Supplementary Figure 9. Lysosomal inhibition suppresses drug-induced cell death in RCC cells silenced of NOX4. **(a)** In parallel to Figure **6c**, total cell lysates were prepared and PARP cleavage was analyzed by Western blot analysis. (**b**) Quantitation of PARP expression from (**a**) is provided**.** The results are expressed as the means using one-way ANOVA with Tukey's post hoc test where ± S.E.M ***p<0.001 shNOX4 etoposide treated compared to shVector (shVec) etoposide treated and ± S.E.M ###p<0.001 shNOX4 etoposide + 3-MA compared to shNOX4 etoposide treated cells.

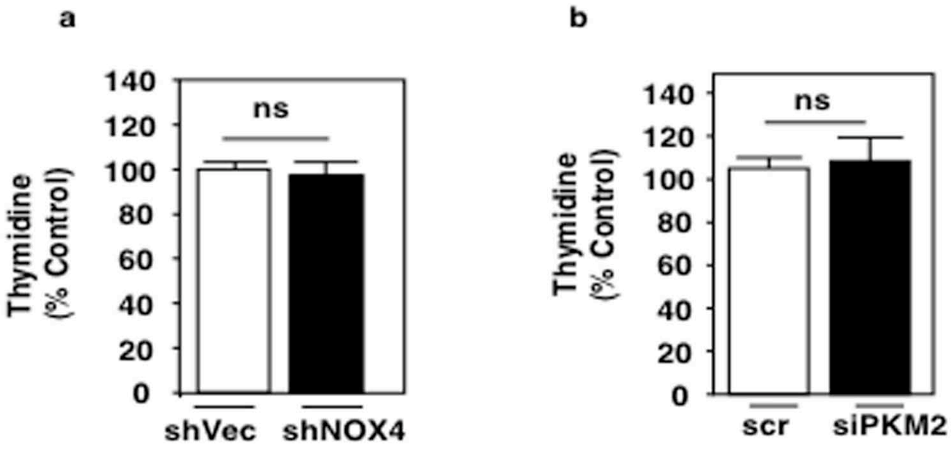

Supplementary Figure 10. Silencing of NOX4 or PKM2 does not reduce cell growth in VHL-deficient cells. (a) DNA synthesis was assessed by [3H]thymidine incorporation in VHL-deficient 786-O cells stably silenced of NOX4 or **(b)** transiently silenced of PKM2. In parallel, shVector (shVec) and scrambled (scr) controls respectively were transfected as described in materials and methods. 3-independent experiments of 3 wells each condition per experiment were assessed and the results expressed as % control using one-way ANOVA with Tukey's post hoc test where the means ± S.E.M *$p<0.05$ compared to shVec control.

a

Protein (amino acid)	MitoProt score (%)
hNOX4 (1-579)	97
hp22phox (1-196)	0.02

b

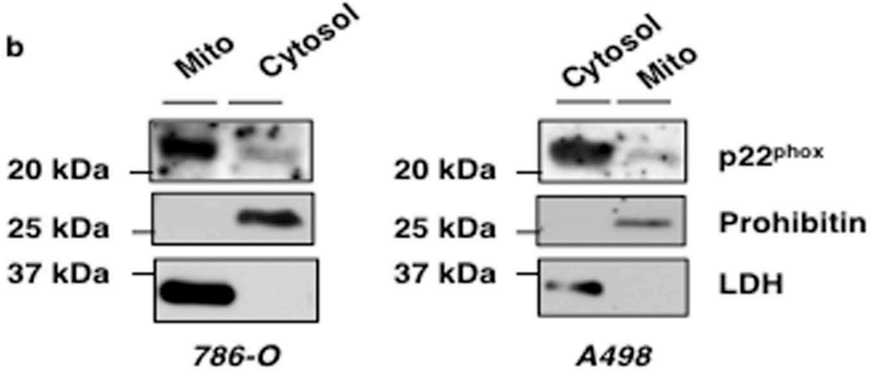

Supplementary Figure 11. Characterization of p22phox's localization to the mitochondria. (**a**) Examination of NOX4 and p22phox sequences to predict probability of localization to the mitochondria using MitoProt prediction software as outlined in materials and methods. (**b**) VHL-deficient RCC cells (786-O and A498) were fractionated into mitochondrial and cytosol fractions using the Pierce kit as described in materials and methods. Western blot analysis was performed using equal protein concentrations for expression of p22phox. LDH was used as a cytosolic marker. Prohibitin was used as a mitochondrial marker.

a

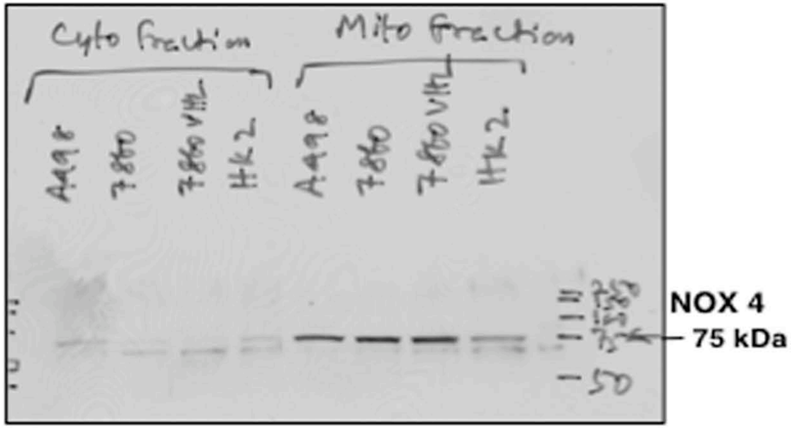

Fig. 2b

b

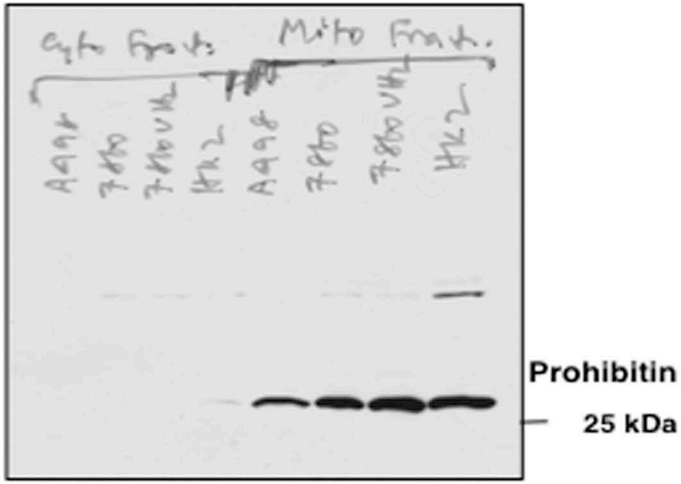

Fig. 2b

Supplementary Figure 12. Uncropped images of Figure 2b showing NOX4 localizes to the mitochondrial compartment. (a) HK2, 786-O (with or without VHL) and A498 cells were subjected to pierce kit fractionation as per manufacturers instructions to yield mitochondrial or cytosolic fractions. NOX4 expression was assessed by Western blot analysis. **(b)** Prohibitin was used as a marker for mitochondria

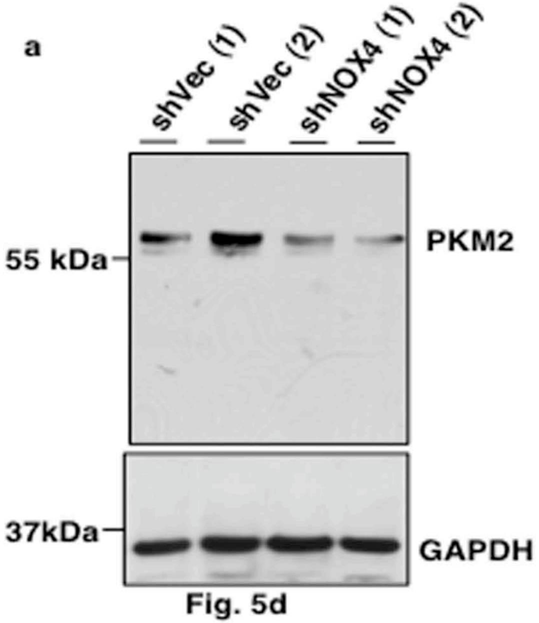

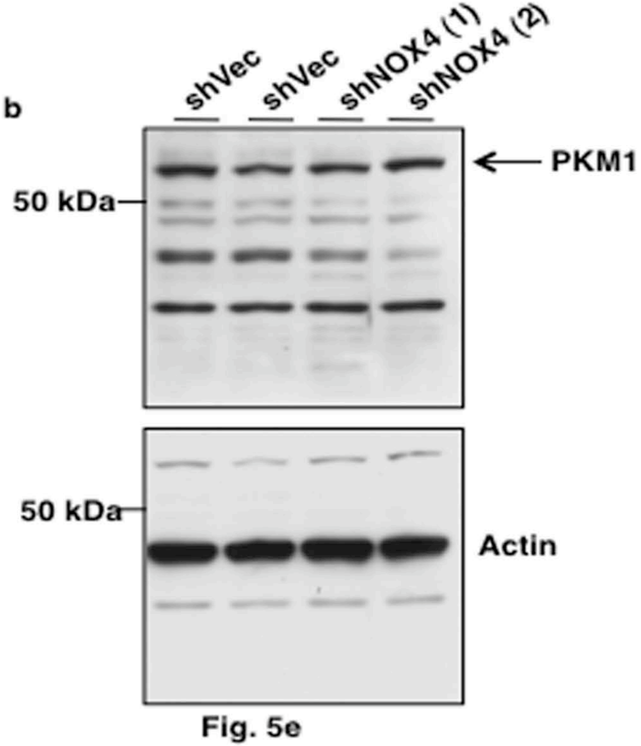

Supplementary Figure 13. Uncropped images of Figures 5d and 5e showing PKM2 and PKM1 expression in NOX4 knockdown RCC 786-O cells. (a) PKM2 or **(b)** PKM1 expression was examined by Western blot in shVector or shNOX4 RCC 786-O stable cell lines. GAPDH and Actin were used as loading controls respectively.

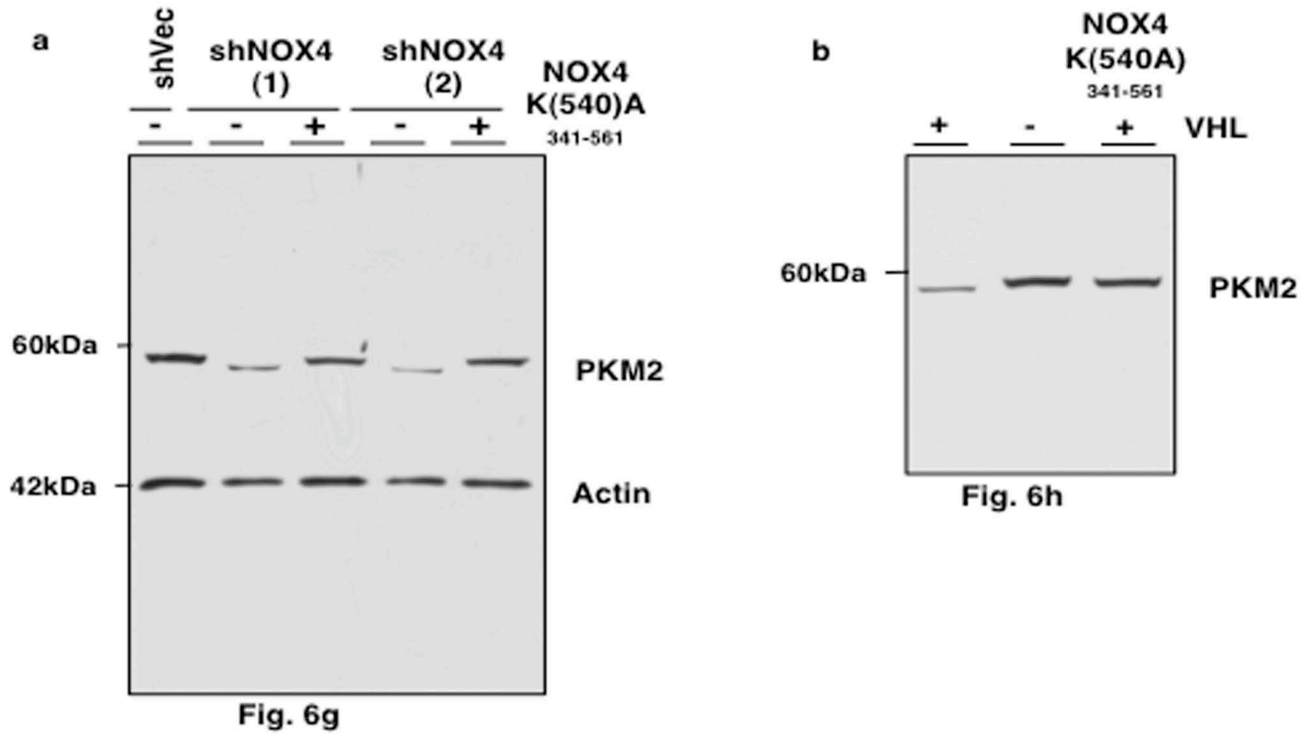

Supplementary Figure 14. Uncropped images of Figures 6g and 6h. (**a**) PKM2 expression was assessed by Western blot analysis in 786-O cells stably silenced of NOX4 or shVector control with transfection of the ATP mutant NOX4 (K540A). (**b**) PKM2 expression was examined in 786-O with or without VHL, transfected (+) or not (-) with ATP mutant NOX4 (K540A). Actin was used as loading control.

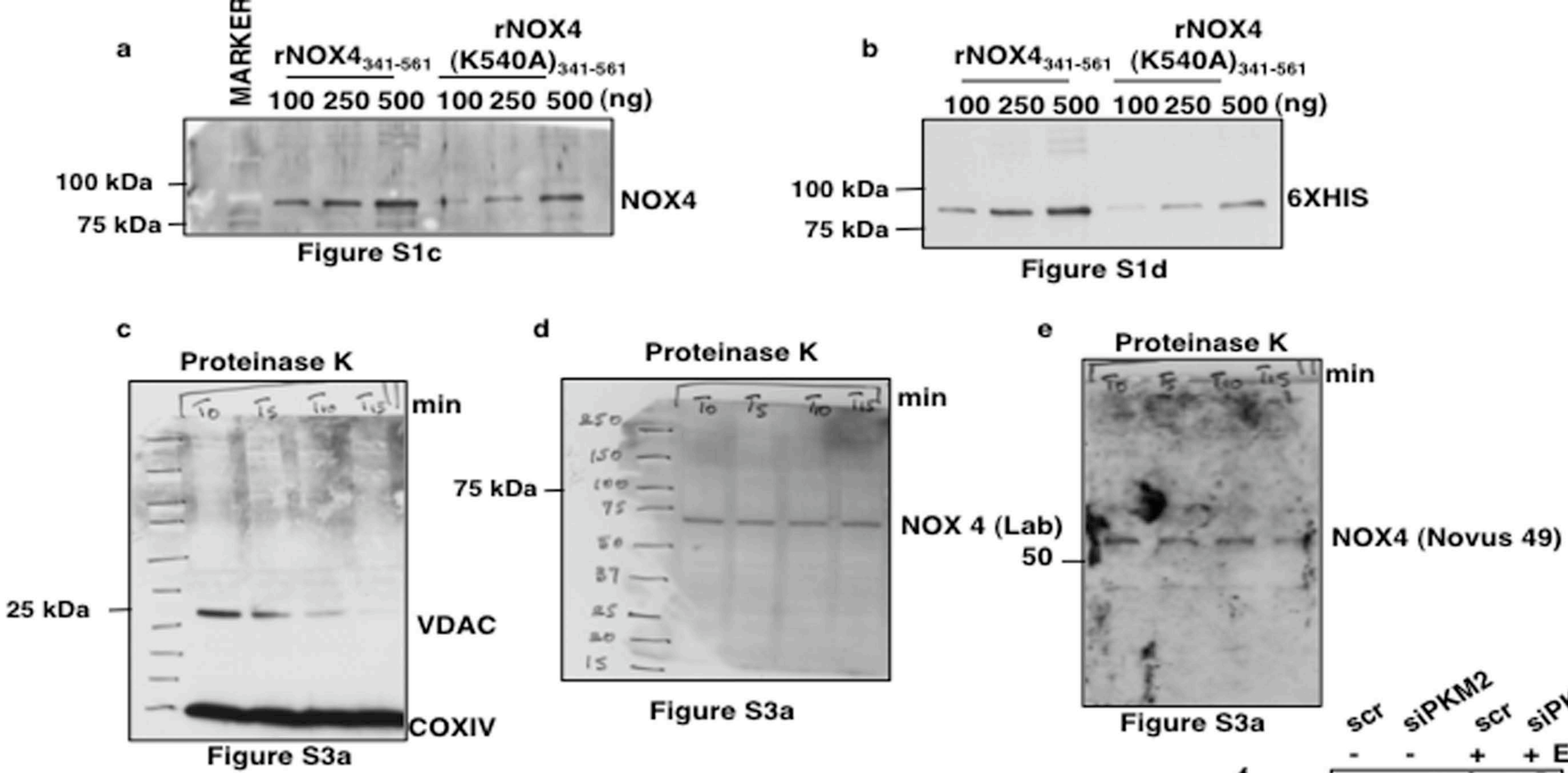

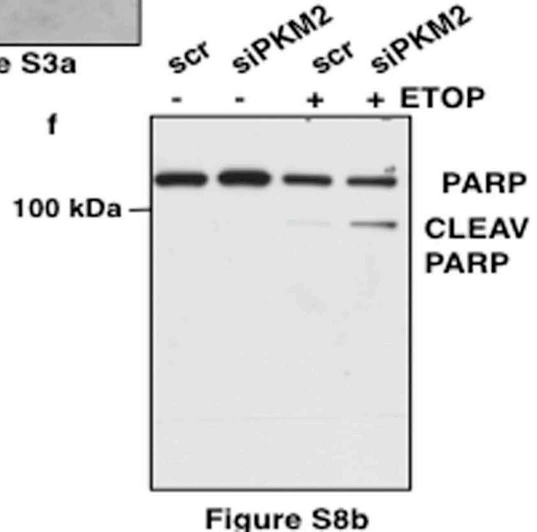

Supplementary Figure 15. Uncropped images of Supplementary Figures S1c, S1d, S3a, and S8b. (**a**,**b**) Increasing amounts of rNOX4$^{341-561}$ and NOX4 (K540A)$^{341-561}$ purified proteins were resolved on two-independent SDS-PAGE gels and Western blot analysis was performed using (**a**) NOX4 antibodies (Lab) and (**b**) 6xHIS (right panel). (**c**,**d**,**e**) Percoll gradient purified mitochondria fraction was prepared from rat kidney cortex and equal amounts of protein were subjected (+) or not (-) to proteinase K digestion over time (T0, T5, T10, and T15 min). (**c**) VDAC, COX VI, and (**d**) NOX4 expression using (Lab) antibody or (**e**) NOX4 (Novus 49) antibody were analyzed by Western blot analysis. (**f**) Scrambled (scr) or siRNA against PKM2 (siPKM2) was transfected in RCC A498 cells. After 48 hours, transfected (Amaxa) cells were incubated (+) with or without (-), etoposide (100 μM) for 14 hours. Cell lysates were prepared and PARP cleavage was examined by Western blot analysis.
